# Glass Transition Behavior of Wet Polymers

**DOI:** 10.3390/ma14040730

**Published:** 2021-02-04

**Authors:** Hai Li, Rui Xiao

**Affiliations:** 1 College of Mechanics and Materials, Hohai University, Nanjing 210098, China; lihai@hhu.edu.cn; 2 Key Laboratory of Soft Machines and Smart Devices of Zhejiang Province, Department of Engineering Mechanics, Zhejiang University, Hangzhou 310027, China

**Keywords:** glass transition, viscoelastic, relaxation spectrum, fractional Zener model

## Abstract

We have performed a systematical investigation on the glass transition behavior of amorphous polymers with different solvent concentrations. Acrylate-based amorphous polymers are synthesized and treated by isopropyl alcohol to obtain specimens with a homogenous solvent distribution. The small strain dynamic mechanical tests are then performed to obtain the glass transition behaviors. The results show that the wet polymers even with a solvent concentration of more than 60 wt.% still exhibit a glass transition behavior, with the glass transition region shifting to lower temperatures with increasing solvent concentrations. A master curve of modulus as a function of frequency can be constructed for all the polymer–solvent systems via the time–temperature superposition principle. The relaxation time and the breadth of the relaxation spectrum are then obtained through fitting the master curve using a fractional Zener model. The results indicate that the breadth of the relaxation spectrum has been greatly expanded in the presence of solvents, which has been rarely reported in the literature. Thus, this work can potentially advance the fundamental understanding of the effects of solvent on the glass transition behaviors of amorphous polymers.

## 1. Introduction

Amorphous polymers exhibit a glass transition behavior, across which the thermomechanical properties, such as modulus, heat capacity, coefficient of thermal expansion, etc., all exhibit a tremendous change. Thus, it is important to investigate glass transition behavior of amorphous polymer systems, which mainly depends on the chemical composition of polymers. However, it can also be affected by other factors, such as aging [1] and plasticization effects [2,3,4,5,6]. 

Plasticizers are known for the ability to modify mechanical behaviors of polymers. By embedding in and distributing through polymer matrix, the plasticizers can separate polymer chains and weaken the intermolecular interaction of polymers, resulting in an increase in mobility of polymer chains and consequently a decrease in glass transition temperature ($T_{g}$) [7,8]. The effects of a plasticizer depend on the concentration as well as its compatibility with the polymer matrix [9]. For example, Da Silva et al. [10] adopted X-ray diffraction, differential scanning calorimetry (DSC) and Fourier transform infrared spectroscopy to characterize the change in mechanical properties of polyvinylchloride (PVC) reinforced with a natural polymeric plasticizer. The plasticizer has good compatibility with the PVC and can considerably modify the mechanical properties of PVC.

Solvent is also widely used as a plasticizer. It has been shown that that solvent can reduce $T_{g}$ of amorphous polymers. For example, Kawai and Hagura [11] investigated the $T_{g}$ of carbohydrate polymer solution systems using DSC. The result showed that the glass transition behaviors of carbohydrate polymer solution systems can be classified into three regions according to the concentration of solution, and that the glass transition region was much broader at an intermediate solution concentration than at high and low concentrations. Huang et al. [2,3,4] found that $T_{g}$ of polyurethane polymers can be reduced dramatically after treatment by water, and the key role behind the phenomenon is that bound water acts as a plasticizer. In addition, Xiao et al. [5] used organic solvents to tune the glass transition region of amorphous polymers. The effect of solvent on the glass transition region has been employed to achieve solvent responsive shape-memory effects, which has received extensive investigation in recent years [2,3,4,12,13,14].

Though it is clear that solvents can play the role of plasticizer to change glass transition behaviors, a comprehensive investigation on the effect of solvents on glass transition behaviors is still lacking. In this work, we investigate the glass transition behaviors of crosslinked amorphous thermosets with different solvent concentrations through the dynamic mechanical analysis. We aim to understand the effects of solvent concentration on the glass transition region as well as the viscoelastic properties across the glass transition region. The paper is arranged as follows. Section 2 presents the methods of material synthesis as well as mechanical characterization. The analysis procedures are also shown in this section. Section 3 shows the main results including the effects of solvent on the glass transition region and the relaxation spectrum. Finally, the findings are summarized in the conclusion part. 

## 2. Experimental Methods

### 2.1. Material Synthesis

The monomer tert-butyl acrylate (tBA), the crosslinker poly (ethylene glycol) dimethacrylate (PEGDMA), with typical molecular weight of 550 and photo-initiator 2,2-dimethoxy-2-phenylacetophenone (DMPA) were ordered from Sigma Aldrich, and isopropyl alcohol (≥99.5%) (IPA) was ordered from Aladdin. All chemicals were used as received. Two different solution were prepared by mixing tBA with PEGDMA, with a weight ratio of 98%:2% or 80%:20%. The photo-initiator, DMPA, was then added to the comonomer solution at a concentration of 0.2 wt.% of the total comonomer weight. The mixture was then injected into two glass slides separated by a 1 mm space and cured in a UV oven (CL-1000L Crosslinker, Analytik Jena, Upland, CA, USA) for 20 min. After the UV curing, the specimens were further thermally cured in an oven at 80 °C for 1 h to achieve a full polymerization. Table 1 lists the polymers synthesized in this work.

As described above, the acrylate-based polymers can be synthesized through one-step photo-polymerization. In addition, the crosslink density, related to the rubbery modulus, and the glass transition region can both be easily tuned in this material system, which makes acrylate-based polymers good candidates as shape-memory polymers [5]. 

### 2.2. Treatment by IPA

Rectangular specimens with a size of 20 mm × 5 mm × 1 mm were used to measure the swelling ratio. Each specimen was weighed before testing. The specimens were immersed in IPA for different times and then taken out of the solvent and wrapped with aluminum foil for three days at room temperature (20–25 °C), then weighed again by a digital balance with an accuracy of 10^−4^ g. This step is to ensure a homogenous distribution of solvents in specimens. For each measurement, three specimens were repeated. The swelling ratio $S_{w}$ is then defined as

(1) $$S_{w}=\frac{m_{t}-m_{0}}{m_{0}}$$

where $m_{0}$ is the weight of initial dry specimen and $m_{t}$ is the weight after treated by IPA.

### 2.3. Dynamic Temperature Sweep Tests

The glass transition region of polymers was measured using a TA Q800 Dynamic Mechanical Analyzer (DMA, TA Instruments, New Castle, DE, USA). The specimens with or without treatment in IPA were subjected to 0.2% dynamic strain with a frequency of 1 Hz and a heating rate of 2 °C/min. For polymers with 2 wt.% crosslink density, the specimens with an immersion time of 30 min, 1, 5 and 10 h were chosen for the dynamic mechanical characterization, while for polymers with 20 wt.% crosslink density, the specimens with an immersion time of 1, 2 and 10 h were chosen.

### 2.4. Dynamic Frequency Sweep Tests

Amorphous polymers exhibit a time-dependent viscoelastic response in the glass transition region, such as stress relaxation, creep and rate-dependent stress responses. The time scale for relaxation may span several decades. Thus, it is difficult to obtain all the characteristics of relaxation within a single test. Fortunately, the time–temperature superposition (TTS) principal works for many amorphous polymers. Thus, it is possible to construct a master curve for viscoelastic responses at one single reference temperature by conducting tests at various temperatures. 

Here the dynamic frequency sweep tests are adopted to obtain the relaxation spectrum of polymers with different solvent concentrations. Before tests, Vaseline was painted on the surface of the wet specimens in order to prevent significant evaporation of solvents at high temperatures. The specimens were heated in a discrete manner with an interval of 5 °C, annealed at each test temperature for 5 min and then subjected to a 0.2% dynamic strain at 0.3, 1, 3, 10 and 30 Hz. 

**Figure 1:**
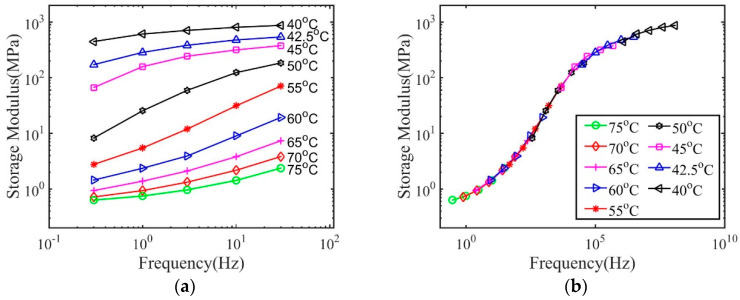
The storage modulus as a function of frequency for dry polymers with 2 wt.% crosslink density (**a**) measured at different temperatures, and (**b**) shifted to form a master curve.

Here, we use the results for dry polymers with 2 wt.% crosslink density as an example to demonstrate the procedures to obtain the master curve and the relaxation spectrum. As shown in Figure 1a, the storage modulus depends on temperature and frequency. The frequency-dependent storage modulus at various temperatures was then shifted to the reference temperature 75 °C through only a horizontal shift to form a master curve shown in Figure 1b. 

**Figure 2:**
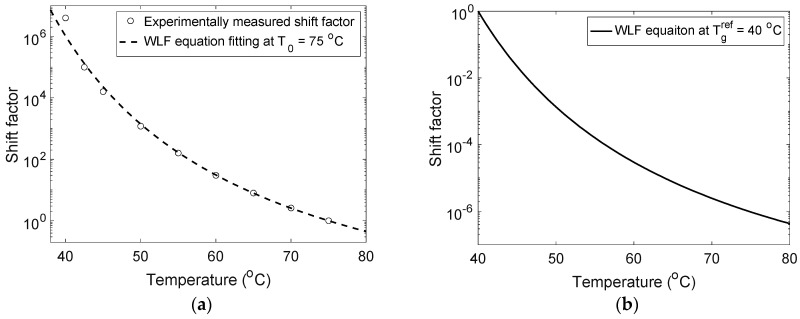
The temperature-dependent shift factor $a_{T}\left(T\right)$ for dry polymers with 2 wt.% crosslink density at (**a**) the reference temperature $T_{0}$ and (**b**) the glass transition temperature $T_{g}^{\mathrm{ref}}$.

The shift factor $a_{T}\left(T\right)$ used to construct the master curve is plotted in Figure 2a. It can also be seen that the shift factor can be fitted by the Williams–Landel–Ferry (WLF) equation [15] with the following form

(2) $$\log\;a\left(T\right)=\;\frac{-C_{1}^{0}\left(T-T_{0}\right)}{C_{2}^{0}+T-T_{0}}$$

where $C_{1}^{0}$ and $C_{2}^{0}$ are the WLF constants at the reference temperature $T_{0}$. The reference temperature $T_{0}$ is close to the end temperature of the glass transition region. The procedures to obtain $C_{1}^{0}$ and $C_{2}^{0}$ can be found in some literatures [15,16]. The WLF constants at $T_{g}^{\mathrm{ref}}$, which correspond to the beginning temperature of the glass transition region, can also be obtained using the following relationship:

(3) $$C_{2}^{g}=C_{2}^{0}+T_{g}^{\mathrm{ref}}-T_{0},\;C_{1}^{g}=\frac{C_{1}^{0}C_{2}^{0}}{C_{2}^{g}}$$

The parameters for WLF constants for polymers with 2 wt.% crosslink density are listed in Table 2, while the corresponding values for polymers with 20 wt.% crosslink density are listed in Table 3. Figure 2b plots the shift factors at the glass transition temperature $T_{g}^{\mathrm{ref}}=40\;℃$, with the obtained values of $C_{1}^{g}$ and $C_{2}^{g}$.

To analyze the effects of solvents on the relaxation behaviors, the master curve of the storage modulus is further fitted by a fractional Zener model [16,17,18]. The Zener model is composed of a spring in parallel with a Maxwell element. The spring is used to represent the equilibrium elastic response while the Maxwell element is used to describe the viscoelastic response. However, it is found that the Maxwell model predicts an exponential form for the relaxation response, which is not consistent with the actual relaxation response in polymers. Alternatively, the integer order derivative of the Maxwell model can be replaced by a fractional order derivative, which provides a better description on the viscoelastic response of polymers. Thus, the fractional Zener model in total contains four parameters: the rubbery modulus $E^{\mathrm{eq}}$, the glassy modulus $E^{\mathrm{neq}}$, the characteristic relaxation time τ, and the fractional order α denoting the breadth of the relaxation spectrum [17]. The analytical expression of storage modulus can be represented as,

(4) $$E_{\mathrm{frac}}^{\prime }\left(\omega \right)=E^{\mathrm{eq}}+\frac{E^{\mathrm{neq}}\left({\left(\omega \tau \right)}^{2\alpha }+{\left(\omega \tau \right)}^{\alpha }\cos\left(\frac{\alpha \pi }{2}\right)\right)}{1+{\left(\omega \tau \right)}^{2\alpha }+2{\left(\omega \tau \right)}^{\alpha }\cos\left(\frac{\alpha \pi }{2}\right)}.$$

The rubbery modulus and the glassy modulus can be obtained from the modulus in the plateau regions. To determine the values of the parameters τ and α, the following function was minimized:

(5) $$Error=\;\sum {\left(\log E\left(\omega \right)-\log E_{\mathrm{frac}}^{\prime }\left(\omega \right)\right)}^{2}$$

where $E_{\mathrm{frac}}^{\prime }\left(\omega \right)$ and $E^{\prime }\left(\omega \right)$ are the fitted and measured storage modulus, respectively.

**Figure 3:**
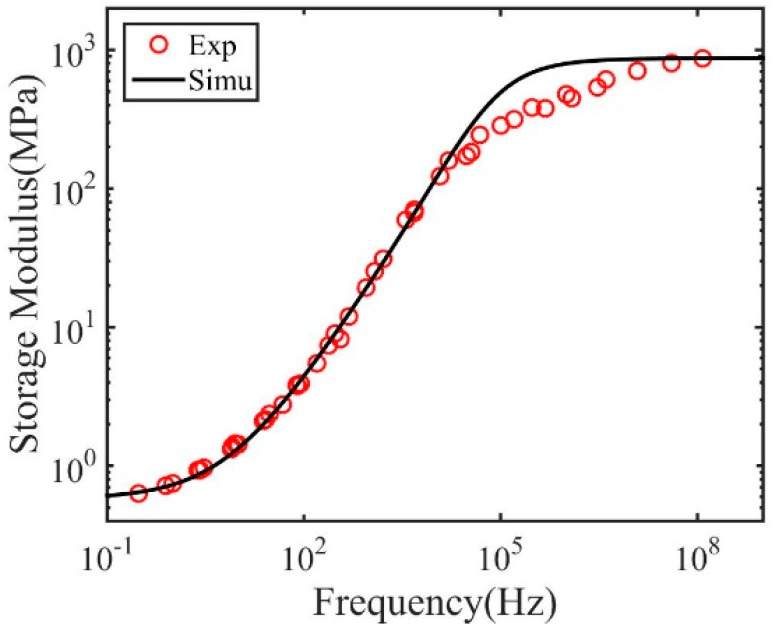
Measured and fitted master curve for dry polymers with 2 wt.% crosslink density.

As shown in Figure 3, the master curve can be well described by the fractional Zener model with the model parameters listed in Table 4 and Table 5. However, at the highest frequency, the fractional Zener model overestimates the storage modulus. This is because the fractional Zener model is based on the equilibrium structure state. At the highest frequency, corresponding to low temperature, the polymer structure may fall out of the equilibrium due to structural relaxation.

## 3. Results and Discussions

**Figure 4:**
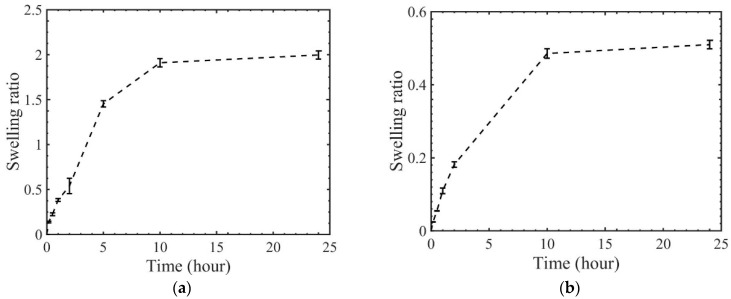
Swelling ratio of acrylate polymer with (**a**) 2 wt.% and (**b**) 20 wt.% crosslink density.

Figure 4 plots the swelling ratio as a function of time. As shown, tBA–co–PEGDMA networks with 2 wt.% and 20 wt.% crosslink density can reach an equilibrium swelling state in around 10 h. The maximum swelling ratio of acrylate copolymer with 2 wt.% and 20 wt.% crosslink density are around 200% and 50% respectively. Polymers with a smaller crosslink density can exhibit a larger swelling ratio, which is as expected. In thermodynamics, more elastic energy of stretching polymer chains is needed for polymers with a denser crosslinked state, which results in a smaller swelling ratio.

**Figure 5:**
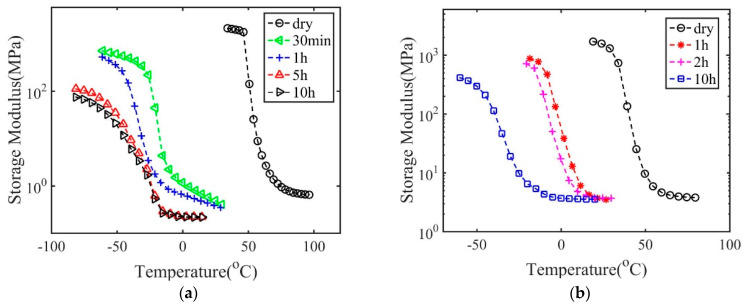
The storage modulus as a function of temperature of (**a**) polymers with 2 wt.% crosslinking density and (**b**) 20 wt.% crosslink density and treatment in isopropyl alcohol (IPA) for different times.

The storage modulus of specimens with different immersion time is shown in Figure 5. All specimens exhibit a glass transition behavior. For polymers with 2 wt.% crosslink density and immersed in IPA for 10 h, the weight fraction of IPA is around 66%. Thus, the material is inherent a polymeric gel. Glass transition can still occur for this wet polymer in the extremely low temperature region from −70 to −20 °C. For all polymers, in the glass transition region the modulus decreases by 2–3 orders as temperatures increases. With increasing the solvent concentration, the glass transition region shifts to the lower temperature region, indicating an increase in the plasticization effects. For both polymers, when immersed in IPA for 10 h, the glass transition region shifts more than 80 °C. For polymers with 2 wt.% crosslink density, a tremendous shift occurs for specimens with immersion time of only 30 min in IPA. This indicates that a small amount of solvent can induce a pronounced plasticization effect. The glass transition region of polymers with 5 and 10 h immersion time in IPA almost overlaps. This is because the plasticization effect saturates with a large amount of solvent. In addition to the glass transition region, both the glassy modulus and rubbery modulus decrease with the increasing solvent concentration.

**Figure 6:**
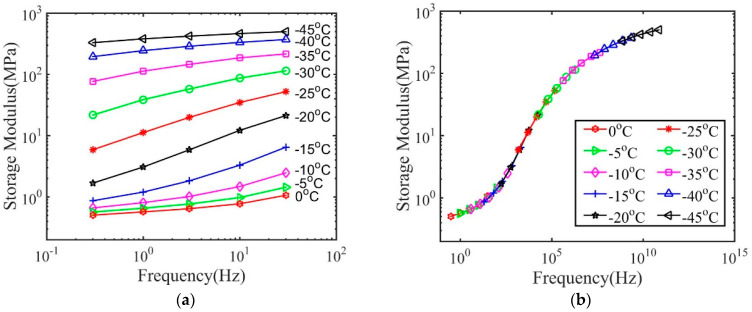

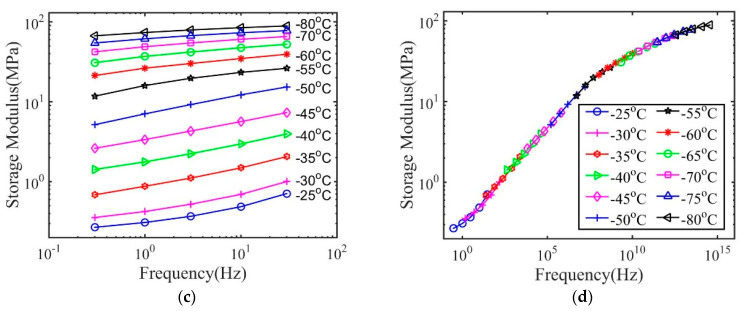
The storage modulus for polymers with 2 wt.% crosslink density and treatment by IPA for (**a**) 1 h and (**c**) 10 h, and the master curve for (**b**) 1 h and (**d**) 10 h.

**Figure 7:**
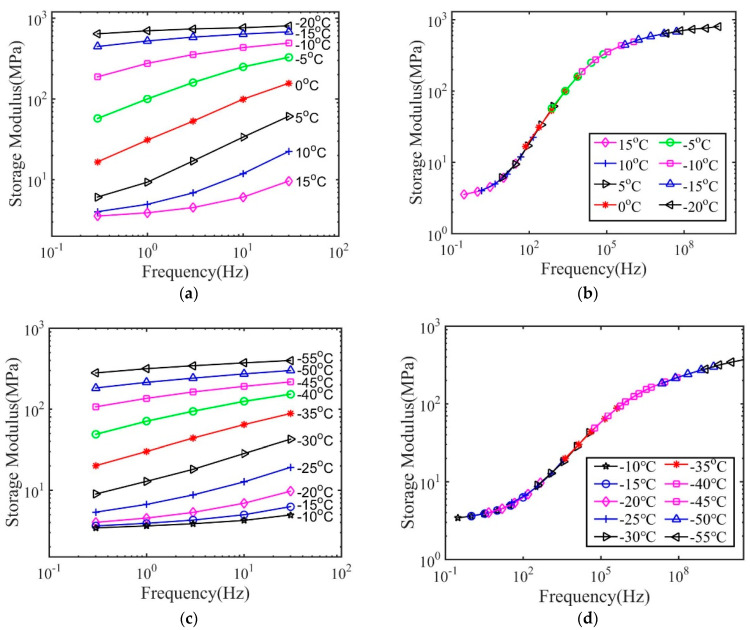
The storage modulus for polymers with 20 wt.% crosslink density and treatment by IPA for (**a**) 1 h and (**c**) 10 h, and the master curve for (**b**) 1 h and (**d**) 10 h.

We then used the method shown in Section 2.4 to construct the master curve for various polymer–solvent systems. The results for polymers with 1 and 10 h immersion time in IPA are shown in Figure 6 and Figure 7 to demonstrate the typical glass transition behaviors of wet polymers. All the other cases show similar behaviors. As shown, the storage modulus of wet polymers also depends on temperature and frequency, which does not show any qualitative difference from that of dry polymers. A master curve can be constructed, which suggests the time–temperature superposition principle can be applied for wet polymers. The master curve of the storage modulus spans a broad frequency region for both dry and wet polymers, indicating that a broad distribution of relaxation time exists.

**Figure 8:**
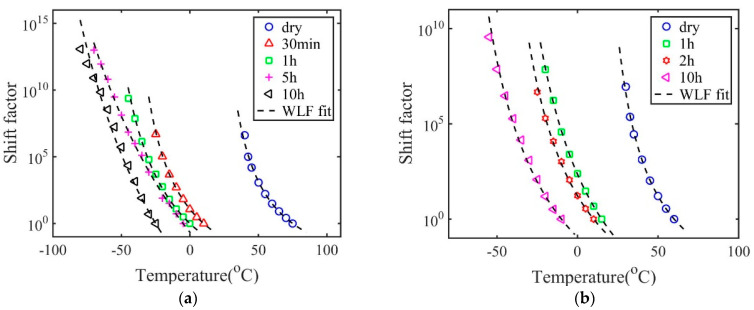
The temperature-dependent shift factor for polymers with (**a**) 2 wt.% and (**b**) 20 wt.% crosslink density.

The procedure to construct the master curve also provides the information for the shift factor $a_{T}\left(T\right)$. In general, the final value of shift factor of dry polymers is several orders smaller than that of wet polymers. As shown in Figure 8, all the shift factors can be well described by the WLF equation. The obtained WLF constants at the reference temperature and the glass transition temperature for polymers with 2 wt.% and 20 wt.% crosslink density are listed in Table 2 and Table 3 respectively. The WLF equation can be derived based on the free volume theory [19,20,21,22,23], which states that the free volume of polymer decreases with a decrease in temperature. The WLF equation has been successfully applied to describe the shifting factor of various material systems, such as polymers, composites and biological materials [15,16,24,25]. Here we show that even for polymer–solvent systems with a large fraction of solvents, the WLF equation can still provide a good description of the shift factors. Thus, the fundamental mechanism of glass transition is the same for the wet polymers and dry polymers. As shown in Table 2 and Table 3, both $T_{0}$ and $T_{g}^{\mathrm{ref}}$ decrease with immersion time, indicating that the glass transition region is shifted to lower temperature regions. The parameter $C_{2}^{g}$ is larger for wet polymers than the dry polymers. This indicates that the Kauzmann temperature is a further departure from the onset glass transition temperature. 

**Figure 9:**
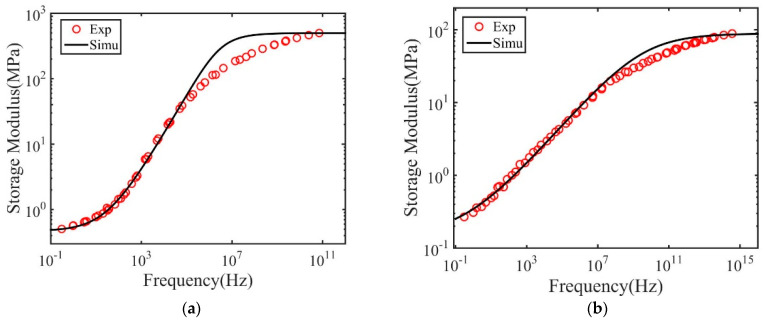
Measured and fitted master curve for polymers with 2 wt.% crosslink density and subjected to (**a**) 1 h and (**b**) 10 h immersion time in IPA.

**Figure 10:**
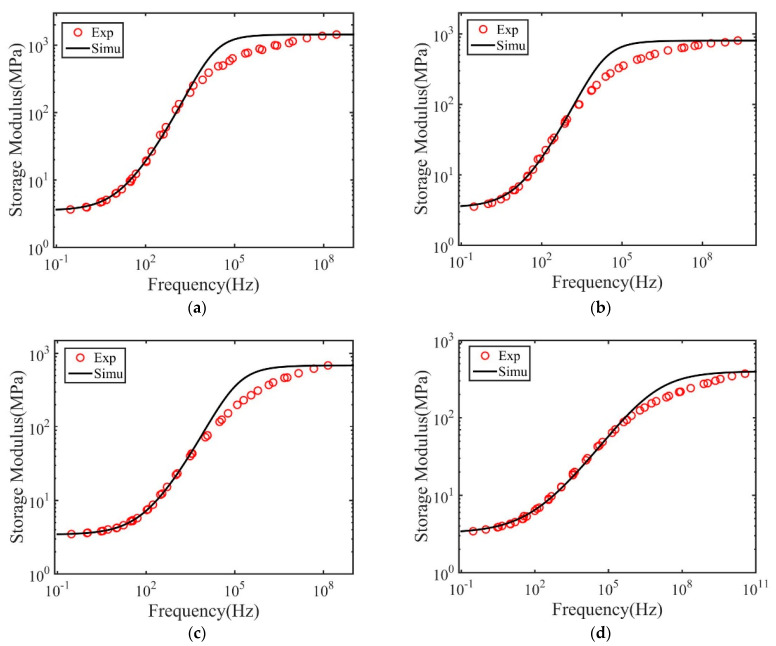
Measured and fitted master curve for polymers with 20 wt.% crosslink density and subjected to (**a**) 0 h, (**b**) 1 h, (**c**) 2 h and (**d**) 10 h immersion time in IPA.

We further analyzed the master curve using the fractional Zener model. Figure 9 and Figure 10 plot the master curves measured from experiments together with the fitted results for both polymers. The obtained model parameters and their significance are listed in Table 4 and Table 5. As shown, the fractional Zener model with four parameters can generally describe the master curve obtained through the time–temperature superposition method. Similar to the results of the dry polymers, it is also found that the model fails to capture the storage modulus in the high-frequency region. This is probably because this region corresponds to the results measured close to the glass transition temperature. The polymer structures may fail out of the equilibrium state due to structural relaxation. In this situation, the measured storage modulus is smaller than that in the equilibrium state. 

The parameters listed in Table 4 and Table 5 can directly reflect the influence of solvents on the glass transition behaviors of polymers. First, both the glassy modulus and rubbery modulus decrease with increasing solvent concentration. The glassy modulus for polymers with 2 wt.% crosslink density decreases by almost 10 times from dry state to fully saturated state. In comparison, the rubbery modulus only changes from 0.58 to 0.15 MPa. For polymers with 20 wt.% crosslink density, the rubbery modulus only slightly changes with solvent. For polymer gels, it has been shown that the rubbery modulus in the wet state is scaled with that of dry state as $E_{w}^{\mathrm{eq}}=E_{d}^{\mathrm{eq}}/J^{1/3}$, where J is the ratio of volume after swelling and that of the initial dry state [26]. The two acrylate-based polymers have a density of around 1.03–1.05 g/cm^3^, while IPA has a density of 0.785 g/cm^3^. Based on the swelling results shown in Figure 4, for acrylate-based polymer with 2 wt.% crosslink density the ratio of the volume in the equilibrium swelling state and the dry state is around 3.7, while that for polymers with 20 wt.% crosslink density is around 1.7. The value of J and the corresponding density of other wet polymers can also be calculated based on the assumption of the volumetric incompressibility for mixture. Thus, the measured value is generally consistent with the above relationship. There are few theories to relate the glassy modulus of the wet polymers with dry polymers. Our results can potentially be used to validate a future developed theory. The value for the relaxation breadth α continuously decreases with increasing solvent concentration for both polymers. A smaller value of α indicates a broader relaxation spectrum. Thus, solvents can expand the breadth of the relaxation spectrum. So far, few works have been performed to investigate the effects of solvent on the relaxation spectrum both in experimental and theoretical aspects. One possible explanation is that extra chemical or physical bonding may be formed between solvent and polymer molecules. The strength of intermolecular interaction in polymers can be further affected by the newly formed bonding, which further results in a change in the relaxation responses [27]. In addition to solvents, some works [28,29] also show that the relaxation spectrum of dry polymers can be changed by mechanical deformation. 

## 4. Conclusions

Glass transition can be affected by many factors, such as thermal treatment and plastination effects. In the past several years, our group has combined experiments and theory to comprehensively investigate the effects of physical aging on the glass transition behaviors in amorphous polymers [30,31]. In this work, we focus on understanding the effects of solvents on glass transition. A series of experiments were carried out to characterize the glass transition of amorphous polymers with different solvent concentrations. It is shown that all polymer–solvent systems investigated exhibit a glass transition behavior, even for the systems with more than 60 wt.% of solvents. The glass transition region shifts to a lower temperature with increasing solvent concentration. The classic methods to analyze the dry polymers, such as the time–temperature superposition principal, can still be applied for wet polymers. Specifically, master curves of storage modulus as a function of frequency can be constructed using the time–temperature superposition principal. The shift factors used to construct the master curves can be well fitted by the WLF equation, which indicates the underlying mechanism for glass transition is the same for wet polymers and dry polymers. An analytical model with four parameters was also used to fit the master curve. It is found that solvents can decrease the rubbery modulus and the glassy modulus. More importantly, the presence of solvents can significantly expand the breadth of the relaxation spectrum, which may be attributed to the change of strength of intermolecular interaction caused by the chemical or physical bonding newly formed between solvent and polymer molecules [27]. This raises more challenges for developing theories for the glass transition behaviors of polymers and polymer–solvent systems [32,33]. 

## Figures and Tables

**Table 1:** The polymer synthesized in this work.

Name of Samples	The Mass Ratio (tBA:PEGDMA:DMPA)
Acrylate-based polymer with 2 wt.% crosslink density	98:2:0.2
Acrylate-based polymer with 20 wt.% crosslink density	80:20:0.2

**Table 2:** Parameters of Williams–Landel–Ferry (WLF) constants for polymers with 2 wt.% crosslink density.

Parameter	Dry	30 min	1 h	5 h	10 h	Physical Significance
$T_{0}$ (℃)	75	10	0	−5	−25	The reference temperature
$C_{1}^{0}$	4.70	5.90	7.78	25.52	24.62	First WLF constant at $T_{0}$
$C_{2\;}^{0}$ (℃)	62.34	64.92	79.47	187.80	144.08	Second WLF constant at $T_{0}$
$T_{g}^{\mathrm{ref}}$ (℃)	40	−25	−45	−70	−80	Glass transition temperature
$C_{1}^{g}$	10.72	12.80	17.94	39.03	39.82	First WLF constant at $T_{g}^{\mathrm{ref}}$
$C_{2}^{g}$ (℃)	27.34	29.92	34.47	122.80	89.08	Second WLF constant at $T_{g}^{\mathrm{ref}}$

**Table 3:** Parameters of WLF constants for polymers with 20 wt.% crosslink density.

Parameter	Dry	1 h	2 h	10 h	Physical Significance
$T_{0}$ (℃)	60	15	10	−10	The reference temperature
$C_{1}^{0}$	5.31	10.14	7.81	8.80	First WLF constant at $T_{0}$
$C_{2\;}^{0}$ (℃)	54.06	79.69	73.84	82.33	Second WLF constant at $T_{0}$
$T_{g}^{\mathrm{ref}}$(℃)	30	−20	−25	−55	Glass transition temperature
$C_{1}^{g}$	11.93	18.08	14.85	19.41	First WLF constant at $T_{g}^{\mathrm{ref}}$
$C_{2}^{g}$ (℃)	24.06	44.69	38.84	37.33	Second WLF constant at $T_{g}^{\mathrm{ref}}$

**Table 4:** Parameters of the fractional Zener model for polymers with 2 wt.% crosslink density.

Parameter	Dry	30 min	1 h	5 h	10 h	Physical Significance
$E^{\mathrm{neq}}$ (MPa)	872	527	498	170	89	The glassy moduli
$E^{\mathrm{eq}}$ (MPa)	0.58	0.51	0.47	0.20	0.15	The rubbery moduli
$\tau \left(10^{-9}s\right)$	2100	2150	80	0.010	0.074	Stress relaxation time at $T=T_{0}$
α	0.7	0.68	0.58	0.26	0.28	Breadth of relaxation spectrum

**Table 5:** Parameters of the fractional Zener model for polymers with 20 wt.% crosslink density.

Parameter	Dry	1 h	2 h	10 h	Physical Significance
$E^{\mathrm{neq}}$ (MPa)	1438	805	686	399	The glassy moduli
$E^{\mathrm{eq}}$ (MPa)	3.53	3.46	3.43	3.25	The rubbery moduli
$\tau \left(10^{-9}s\right)$	9270	9200	1690	30	Stress relaxation time at $T=T_{0}$
α	0.72	0.66	0.66	0.42	Breadth of relaxation spectrum

## Data Availability

The raw/processed data required to reproduce these findings cannot be shared at this time as the data also forms part of an ongoing study.
